# When Precision Matters: Bone Marrow Cytology Meets qPCR in a Pilot Study Quantifying *Leishmania infantum* Load in Dogs

**DOI:** 10.3390/microorganisms13092211

**Published:** 2025-09-21

**Authors:** Juliana Costa, Ana Rita Silva, Filipe Sampaio, Ana Patrícia Alves, Hugo Silva, Hugo Lima de Carvalho, Carlos Sousa, Manuel Simões, Cátia Fernandes, Ana Patrícia Lopes, Andreia Garcês, Elsa Leclerc Duarte, Ana Cláudia Coelho, Luís Cardoso, Ricardo Lopes

**Affiliations:** 1 Department of Chemical and Biological Engineering (DEQB), Faculty of Engineering, University of Porto (FEUP), Rua Dr. Roberto Frias, 4200-465 Porto, Portugal; up202208522@up.pt; 2 Molecular Diagnostics Laboratory, Unilabs Portugal, Centro Empresarial Lionesa Porto, Rua Lionesa, 4465-671 Leça do Balio, Portugal; ana.rita.silva@unilabs.com (A.R.S.); ana.tapado.alves@unilabs.com (A.P.A.); hugo.silva@unilabs.com (H.S.); carlos.sousa@unilabs.com (C.S.); 3 CEDIVET Veterinary Laboratories, Lionesa Business Hub, R. Lionesa 446 C24, 4465-671 Leça do Balio, Portugal; filipe.sampaio@cedivet.pt (F.S.); hugo.carvalho@cedivet.pt (H.L.d.C.); 4 Cytology and Haematology Diagnostic Services, Laboratory of Histology and Embryology, Department of Microscopy, ICBAS-School of Medicine and Biomedical Sciences, University of Porto (U.Porto), Rua de Jorge Viterbo Ferreira, 228, 4050-313 Porto, Portugal; 5 LEPABE—Laboratory for Process Engineering, Environment, Biotechnology and Energy, Faculty of Engineering, Department of Chemical and Biological Engineering, University of Porto (U.Porto), 4200-465 Porto, Portugal; mvs@fe.up.pt; 6 ALiCE—Associate Laboratory in Chemical Engineering, Faculty of Engineering, University of Porto (U.Porto), 4200-465 Porto, Portugal; 7 Anicura Santa Marinha Veterinary Hospital, R. Dom Henrique de Cernache 183, 4400-625 Vila Nova de Gaia, Portugal; catia.fernandes@anicura.pt; 8 Department of Veterinary Sciences, University of Trás-os-Montes e Alto Douro (UTAD), Quinta de Prados, 5001-801 Vila Real, Portugal; aplopes@utad.pt (A.P.L.); accoelho@utad.pt (A.C.C.); 9 Animal and Veterinary Research Centre (CECAV), Associate Laboratory for Animal and Veterinary Sciences (AL4AnimalS), University of Trás-os-Montes e Alto Douro (UTAD), Quinta de Prados, 5001-801 Vila Real, Portugal; andreiamvg@gmail.com; 10 Wildlife Rehabilitation Centre (CRAS), Veterinary Teaching Hospital, University of Trás-os-Montes e Alto Douro (UTAD), 5000-801 Vila Real, Portugal; 11 Department of Veterinary Medicine, School of Science and Technology, University of Évora, Polo da Mitra, Apartado 94, 7002-554 Évora, Portugal; emld@uevora.pt; 12 Mediterranean Institute for Agriculture, Environment and Development (MED), Global Change and Sustainability Institute (CHANGE), University of Évora, Polo da Mitra, Apartado 94, 7002-554 Évora, Portugal; 13 Department of Veterinary and Animal Sciences, University Institute of Health Sciences (IUCS), CESPU, 4585-116 Gandra, Portugal

**Keywords:** bone marrow, canine leishmaniosis, clinical pathology, digital cytology, One Health, precision medicine, qPCR, zoonosis

## Abstract

*Leishmania infantum* is the causative agent of canine leishmaniosis (CanL), a zoonotic disease of considerable clinical and epidemiological concern. Quantification of parasite load is valuable for clinical management, particularly in low-parasite-load cases. This pilot study evaluated the correlation between cytological and molecular techniques in bone marrow samples from dogs clinically affected with leishmaniosis. Amastigotes were quantified by two independent observers using light microscopy, and the same samples were analysed by real-time polymerase chain reaction (qPCR) employing *Leishmania* spp. specific commercial primers. Inter-observer agreement was moderate according to Cohen’s kappa (κ = 0.47) and substantial according to the intraclass correlation coefficient (ICC = 0.63), respectively, confirming measurement reproducibility. A very strong inverse correlation was found between parasite counts and qPCR cycle threshold (Ct) values (Spearman’s ρ = −0.89; *p* < 0.001). Furthermore, a robust logarithmic relationship (amastigotes/μL = 10^(−0.158 × Ct + 7.61)^; R^2^ = 0.99994) was established allowing direct estimation of in vivo parasite concentration from molecular data. These preliminary findings suggest that qPCR can serve as a reliable, semi-quantitative tool, offering higher sensitivity in subclinical infections. The integration of molecular quantification with cytological methods enhances diagnostic accuracy and supports personalised therapeutic decision-making, advancing clinical management of CanL in a One Health context.

## 1. Introduction

Canine leishmaniosis (CanL) is a vector-borne disease of global importance, caused mainly by the zoonotic protozoan parasite *Leishmania infantum*. Endemic in 90 countries, its prevalence is particularly pronounced in the Mediterranean basin, including Portugal, where infection and disease remain a persistent veterinary and public health concern [1,2,3]. Over 20 species of the genus *Leishmania* are known to infect humans. Domestic dogs act as the primary urban and peri-urban reservoirs of *L. infantum*, thereby playing a pivotal role in the parasite’s transmission dynamics [4,5,6]. Additionally, other domestic animals can also be infected in areas of endemicity; for example, felines in the Mediterranean basin are also susceptible to leishmaniosis [7]. CanL has also been reported from geographically isolated settings (e.g., Lampedusa island, Italy), highlighting the widespread and multi-host nature of *Leishmania* in this region [8].

The parasite’s life cycle alternates between two distinct morphological stages: the extracellular promastigote, bearing an external flagellum within the phlebotomine sand fly vector, and the intracellular amastigote, which possesses a non-externalised flagellum and resides within macrophages of the vertebrate host [9]. Transmission occurs mainly through the bite of infected *Phlebotomus* spp., which introduce promastigotes into the host’s dermis. These are rapidly phagocytosed by macrophages and transform into amastigotes, which may multiply and disseminate to lymphoid organs, including the bone marrow, as well as secondary lymphoid organs such as lymph nodes and the spleen. Chronic infection may facilitate the return of amastigotes to cutaneous sites, thereby increasing the likelihood of vector acquisition and sustaining the transmission cycle [10,11,12].

Given the frequently subclinical or non-specific presentation of canine infection with *L. infantum*, early and accurate diagnosis is essential for both clinical management and epidemiological control [13]. While conventional cytology, entailing the microscopic identification of amastigotes in bone marrow aspirate smears, remains a standard diagnostic tool [1,14], its sensitivity significantly declines in low-parasite-load cases or suboptimal specimens [15]. Consequently, molecular diagnostics, particularly real-time polymerase chain reaction PCR (qPCR), have emerged as pivotal diagnostic methods, for their higher sensitivity, specificity, and ability to quantify parasite burden. These molecular assays can be applied to various clinical samples, including blood and skin biopsies [16,17].

This study aims to evaluate the possibility of qPCR as a semi-quantitative diagnostic tool for estimating *Leishmania* spp. burden in canine bone marrow samples, by investigating its correlation with conventional cytological analysis. As a pilot study, it seeks to establish a foundational correlation between the number of amastigotes identified microscopically and the corresponding qPCR Ct values, derived from standard curves based on known plasmid concentrations. This exploratory pilot study is intended as a preliminary step toward the validation of molecular quantification techniques in future large-scale clinical and epidemiological studies. All aims are positioned as hypothesis-generating and will require validation in larger and more representative sample sets.

This pilot study pursued three aligned aims: (i) to derive and present a preliminary Ct-to-parasite-burden conversion equation based on paired cytology and plasmid-standardised qPCR in canine bone marrow; (ii) to evaluate inter-observer reliability in cytological quantification; (iii) to explore, rather than validate, the potential of Ct values as a clinically meaningful proxy of tissue parasite burden, and derive a preliminary predictive logarithmic equation to estimate in vivo parasite load.

## 2. Materials and Methods

### 2.1. Study Design and Sample Collection

This study employed an analytical design to evaluate the correlation between cytological amastigote quantification and qPCR-derived Ct values in bone marrow samples from three dogs naturally infected with *Leishmania*, diagnosed at Veterinary Teaching Hospital of the University of Trás-os-Montes e Alto Douro (UTAD), Vila Real, Portugal, through routine clinical procedures.

All three dogs were clinically affected with confirmed CanL, exhibiting typical signs such as lymphadenopathy, dermatological lesions, and weight loss, consistent with active infection. They were middle-aged (3–6 years old) mixed-breed dogs (two males, one female) with moderate clinical disease (LeishVet stage BII), and no other major co-morbidities.

All clinical procedures complied with the Portuguese legislation for the protection of animals used for scientific purposes (i.e., Decree-Law no. 113/2013, of 7 August 2013), which transposes European legislation (i.e., Directive 2010/63/EU of the European Parliament and of the Council, of 22 September 2010). All samples were leftovers from clinical procedures, which under the oral informed consent of dog tutors, were preserved at the Laboratory of Parasitology, Department of Veterinary Sciences, UTAD before being transferred to CEDIVET Veterinary Laboratories (Porto, Portugal). This workflow ensured that all samples remained properly preserved and traceable from collection through cytological and molecular evaluation.

### 2.2. Sample Collection Procedure

Aspirates were for clinical purposes obtained aseptically from the ribs (6th to 9th), a commonly used site in dogs for bone marrow collection due to accessibility and sample quality. Dogs were positioned in lateral recumbency, and the collection site was clipped and surgically disinfected. Local anaesthesia was administered by infiltrating lidocaine from the skin down to the periosteum to minimise discomfort. Using a sterile bone marrow aspiration needle (18 gauge), the needle was inserted through and advanced with a rotating motion until it penetrated the cortical bone and entered the medullary cavity. Once properly positioned, the stylet was removed and a syringe attached to aspirate the marrow, collecting 1 mL of sample [18].

### 2.3. Sample Handling and Preservation

Immediately after aspiration for clinical diagnostic purposes, the bone marrow sample was transferred into tubes containing EDTA to prevent coagulation and gently mixed. Following the completion of routine diagnostic procedures, surplus material was divided into two aliquots: one destined for cytological analysis (prepared as direct smears on glass slides and air-dried) and another reserved for molecular analysis. The aliquot intended for molecular purposes was promptly placed in sterile microtubes and stored at −40 °C to preserve nucleic acid integrity until DNA extraction and qPCR processing. These leftover samples remained frozen at −40 °C until their use in the present study. For molecular analysis, 200 µL of EDTA-stabilised bone marrow aspirate per sample were reserved for DNA extraction.

### 2.4. Cytological Evaluation

Cytological smears were prepared using standard haematological techniques. A 20 µL aliquot of bone marrow aspirate, stabilised in EDTA, was placed near the frosted end of a glass slide and allowed to flow gently by gravity to promote the deposition of bone marrow spicules, small aggregates of hematopoietic tissue indicative of sample quality and marrow microarchitecture. Using a second slide held at a 30–45° angle, the sample was spread smoothly to create a monolayer smear, essential for optimal microscopic evaluation. Slides were air-dried with gentle manual agitation, avoiding direct heat to preserve cellular morphology.

After drying, slides were stained with May-Grünwald Giemsa (MGG) using an automated RAL Stainer (RAL Diagnostics, Martillac, France), ensuring consistent and reproducible staining quality. Microscopic analysis was performed at 1000× magnification using a Leica DM750 microscope (Leica Microsystems, Wetzlar, Germany) equipped with a HI PLAN 100×/1.25 oil immersion objective. Amastigotes were identified as free or intracellular, spherical, flagellated organisms measuring 2–4 µm in diameter. Slides were digitally scanned using the Philips IntelliSite Pathology Solution 3.2 (Philips Digital & Computational Pathology, Best, The Netherlands) system for inter-observer evaluation and archival purposes. Two experienced observers independently counted amastigotes in triplicate, and inter-observer agreement was assessed to ensure accuracy and reproducibility of quantification. To enhance reproducibility and ensure applicability in routine diagnostic settings, cytological quantification was expressed as the mean number of amastigotes per high-power field (HPF; 1000×), rather than as a total count per slide. Although digital scans (Philips IntelliSite Pathology Solution 3.2) were employed for archiving and illustrative purposes, all quantitative analyses were performed on HPF sampling (20 fields per smear; three smears per case; 60 HPFs per sample), closely reflecting routine cytology workflows.

### 2.5. Molecular Analysis

For each bone marrow sample, a single genomic DNA extraction was performed from 200 µL of EDTA-stabilised aspirate using the Promega AX9760 protocol (Promega Corporation, Madison, WI, USA) for whole blood, adapted for complex cellular matrices such as bone marrow. The process was automated using the KingFisher^TM^ Flex Purification System (Thermo Fisher Scientific, Waltham, MA, USA), with all reagents handled at room temperature (15–30 °C) and according to the manufacturer’s specified conditions.

The extraction workflow was conducted according to the manufacturer’s instructions. An internal extraction control (IEC) was added to each sample to monitor the efficiency and integrity of the process.

Extracted DNA was not quantified prior to real-time PCR, and no inter-sample normalisation (e.g., to total DNA mass or a host reference gene) was performed in this pilot study. Instead, a fixed input of 5 µL of extracted DNA was used per reaction for all samples, thereby standardising the reaction template volume while the IEC monitored extraction performance.

Subsequent molecular detection of *Leishmania* spp. DNA was performed using the NZYtech *Leishmania* spp. PCR Kit^®^ (NZYtech, Lisbon, Portugal). This real-time PCR assay targets conserved regions of the parasite’s genome, including kinetoplast DNA (kDNA), and was designed to maximise specificity and sensitivity through in silico alignment against NCBI reference sequences. Notably, this assay targets conserved *Leishmania* kinetoplast DNA at the genus level. However, since *L. infantum* is the only endemic *Leishmania* species in Portugal (and the causative agent of CanL in the country), any *Leishmania* DNA detected in our samples was presumed to originate from *L. infantum*.

For target quantification the *Leishmania* spp. Quantitative qPCR Standard Kit^®^ (NZYtech, Lisbon, Portugal) was used. This kit is specifically designed for high-sensitivity quantitative qPCR applications. It includes a DNA fragment derived from *Leishmania* species, precisely quantified at 2.0 × 10^6^ genome copies/μL. To enable absolute quantification, a standard curve was generated by preparing a series of serial dilutions of the quantitative standard, each with a defined number of genome copies per microlitre. These dilutions were run in parallel with the test samples during the qPCR assay. The resulting Ct values were plotted against the logarithm of the known concentrations to construct the standard curve, which served as a reference for determining the parasite burden in each test sample.

Real-time qPCR was chosen due to its recognised status as the gold standard in molecular diagnostics [19,20,21,22], offering outstanding accuracy, specificity, and sensitivity for the detection and quantification of *Leishmania* spp. DNA [23,24].

PCR reactions were assembled with 15 µL of master mix and 5 µL of extracted DNA per well, including positive and negative controls. Reactions were loaded into a 96-well plate, sealed with optical adhesive film, centrifuged briefly to eliminate air bubbles, and processed on a QuantStudio^TM^ 5 Real-Time PCR System (Thermo Fisher Scientific, Waltham, MA, USA) following the thermal cycling conditions suggested in the manufacturer’s protocol (Table 1).

Each qPCR run incorporated a positive control (the kit-supplied *Leishmania* DNA standard of known concentration) and a non-template negative control (NTC; nuclease-free water) to verify assay performance.

Fluorescence was monitored in real time through a fluorogenic probe system comprising a 5′ reporter dye and 3′ quencher. During the extension phase, probe cleavage by Taq polymerase resulted in increased fluorescence, directly proportional to the amount of amplified target.

For the final validation of the results, the following criteria had to be met:

- Amplification of the internal extraction control;
- No amplification of the negative control;
- Amplification of the positive control.

Only results meeting these criteria were validated as negative (no amplification of the target), positive (true amplification of the target, indicated by a sigmoidal curve), or invalid (if there was no amplification of the target and no amplification of the internal extraction control, and therefore excluded from the study) [25].

Data interpretation and analysis were performed using the Design & Analysis 2 (DA2) software, version 1.5.3 (Thermo Fisher Scientific, Waltham, MA, USA), in accordance with the manufacturer’s recommendations. The software was employed to extract both qualitative and quantitative parameters, including Ct values and amplification curve profiles, based on predefined analytical settings provided by the manufacturer.

### 2.6. Statistical Analysis

All data were organised in Microsoft Excel^®^ (Microsoft Corporation, Redmond, WA, USA), and statistical analyses were performed using JMP^®^ software version 14.3 (SAS Institute Inc., Cary, NC, USA, 1989–2023) and the DATAtab^®^ online statistical platform (DATAtab e.U., Graz, Austria, 2025).

Descriptive statistics were processed for all quantitative variables, including measures of central tendency (mean, median), dispersion (standard deviation), and distribution shape. Normality of the variables was assessed using the Shapiro–Wilk, Kolmogorov–Smirnov with Lilliefors correction, and Anderson-Darling tests. Given that the data did not conform to the assumptions of normality (*p* < 0.05), non-parametric statistical methods were applied [26].

Inter-observer agreement in cytological quantification of *Leishmania* spp. amastigotes was evaluated using both the intraclass correlation coefficient (ICC) and Cohen’s kappa statistic. The parasite load was categorised as an ordinal variable according to predefined thresholds: absent (0 amastigotes), low (1–10), moderate (11–20), and high (>20 amastigotes per field). Agreement was further examined through Bland–Altman plots to assess systematic bias and potential trends between observers.

The relationship between the mean cytological parasite count (expressed as amastigotes per µL) and the qPCR Ct values was analysed using Spearman’s rank correlation coefficient, due to non-normal distribution of the variables. In addition, Pearson’s correlation coefficient was also calculated to complement the non-parametric analysis.

To estimate parasite burden based on qPCR Ct values, a simple linear regression model was fitted. This model was constructed using plasmid-based standard curves, allowing for extrapolation of in vivo parasite burden from molecular amplification data. Given the small dataset, standard errors and confidence intervals around slope/intercept are expectedly unstable and are therefore interpreted qualitatively. No claims of external validity or clinical decision thresholds are made. Statistical significance was established at a threshold of *p* < 0.05 [26].

## 3. Results

### 3.1. Cytological Quantification of Amastigotes

Cytological evaluation was performed on three bone marrow smears per individual (BM-C1, BM-C2 and BM-C3), with 20 high-power fields (HPFs; 1000×), yielding a total of 60 fields per sample. Amastigote counts were independently conducted by two experienced observers with certified training in clinical pathology. Table 2 presents the mean amastigote counts per field, standard deviation, minimum, maximum, and confidence intervals for each sample and observer.

**Figure 1:**
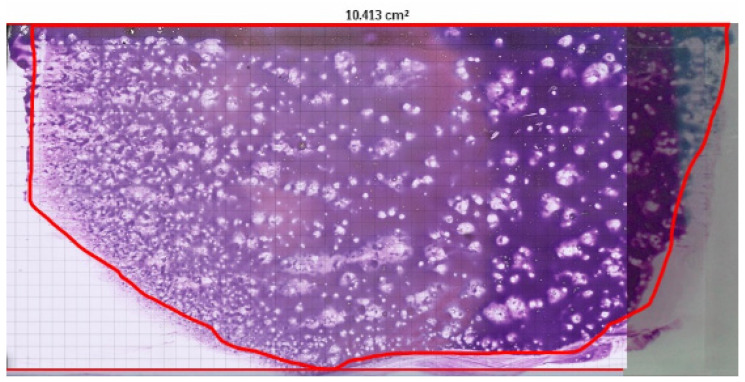
Digital scan of canine bone marrow smear obtained using Philips IntelliSite Pathology Solution 3.2 (Philips Digital & Computational Pathology, Best, The Netherlands). The glass slide measures 76 × 26 mm. The area manually delineated in red represents the region analysed microscopically. The software provided an estimated smear surface area of 10.413 cm^2^, used in the calculation of amastigote concentration per microlitre.

**Figure 2:**
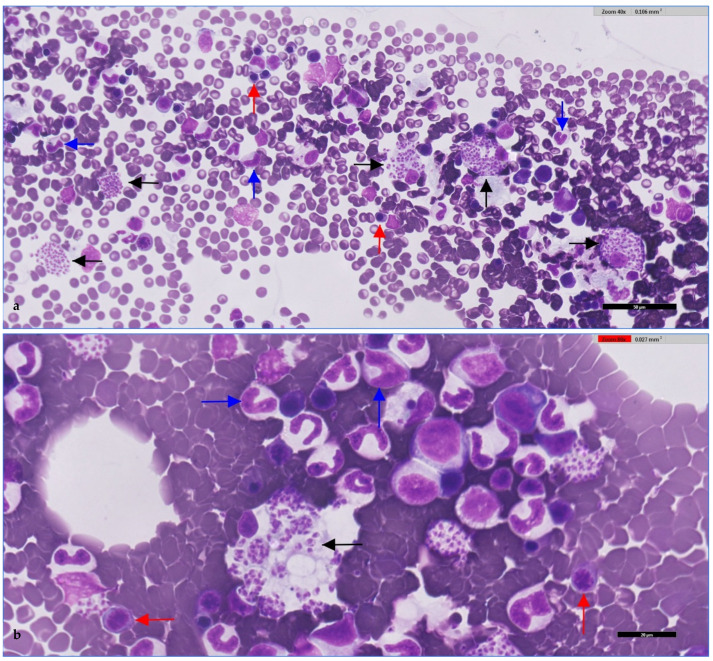
**Canine bone marrow smear.** (**a**) Erythroid precursors (red arrows), granulocytic precursors (blue arrows), and *Leishmania* amastigotes phagocytosed by macrophages (black arrows) within the cytoplasm of macrophages. (**b**) Higher magnification showing amastigotes (black arrows) as small, intensely basophilic, round-to-oval bodies with a distinct nucleus and kinetoplast. Surrounding cells correspond to erythroid (red arrows) and myeloid precursors (blue arrows). qPCR performed in parallel detected *Leishmania* DNA in the sample, confirming that the amastigotes observed cytologically were *Leishmania* spp. (presumptively *L. infantum*). Stained with May-Grünwald-Giemsa (MGG) and digitally scanned using Philips IntelliSite Pathology Solution 3.2 (Philips Digital & Computational Pathology, Best, The Netherlands). Scale bars: 50 μm (**a**); 20 μm (**b**).

Digital cytology and microscopic visualisation revealed intracellular and free amastigote forms, often associated with macrophages. Representative images are shown in Figure 1 and Figure 2.

To estimate the number of amastigotes per microlitre (µL) of bone marrow from direct microscopic observation using 20 µL, the following technical parameters were considered:

- **Total estimated slide area (A):** 10.35 cm^2^.
- **Area of a single microscopic field at 1000× magnification (Ac):** 31,416 µm^2^, calculated using a 100× objective lens and an eyepiece with a field number (FN) of 20 mm.
- **Number of theoretical microscopic fields on the slide (Nc):**

(1) $$Nc=\frac{A\times 10^{8}{µm}^{2}/{cm}^{2}}{Ac}=\frac{{10.35\;cm}^{2}\times 10^{8}\;{µm}^{2}/{cm}^{2}}{31,416\;{µm}^{2}}\approx 32,929\;fields$$

Since the total sample volume was uniformly distributed over the slide, the volume represented by each microscopic field (Vc) was calculated as follows:

(2) $$Vc=\frac{20\;µL}{Nc}\frac{20\;µL}{32,929\;fields}\approx 0.000607\;µ\frac{L}{field}\;$$

The concentration of amastigotes per µL (C) was then estimated using the following formula:

(3) $$C=\frac{Mean\;amastigotes\;per\;field}{Vc}$$

### 3.2. qPCR Amplification and Molecular Quantification

**Figure 3:**
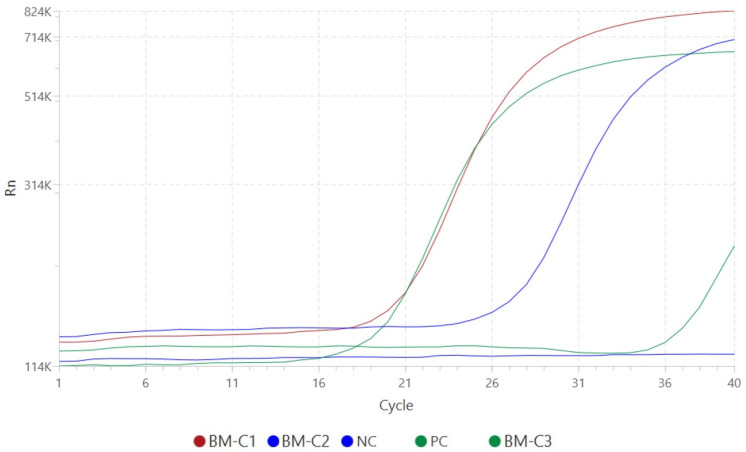
Amplification curves generated by the QuantStudio^TM^ 5 Real-Time PCR System for the detection of *Leishmania* spp. DNA in three bone marrow samples used in this study. Fluorescence signal (Rn, linear scale) is plotted against the number of amplification cycles. Positive amplification is observed in samples BM-C1 (red), BM-C2 (blue), and BM-C3 (dark green), with Ct values of 19.54, 26.00, and 36.59, respectively. The positive control (PC, light green) shows robust amplification, while the negative control (NC, navy blue) exhibits no amplification signal, confirming the specificity and reliability of the assay. The ΔRn was automatically set by the equipment software, corresponding to the point at which the fluorescence exceeds the baseline fluorescence and enters the exponential amplification phase. The Ct values were determined based on this point.

Real-time PCR confirmed the presence of *Leishmania* spp. DNA in all three samples. Amplification plots demonstrated clear, exponential fluorescence curves with Ct values of 19.54 (BM-C1), 26.00 (BM-C2), and 36.59 (BM-C3), indicating varying parasite loads. Positive and negative controls yielded expected outcomes, validating assay integrity. Internal control Ct values (range: 27.33–32.88) were within the acceptable limits, confirming extraction and amplification efficiency (Figure 3).

Even in the cytologically inconclusive case (BM-C3), qPCR detected low levels of *Leishmania* DNA, highlighting its diagnostic sensitivity (Table 3).

Detailed individual-level data, including demographic and clinical details, cytology findings, and qPCR results, are provided as Supplementary Table S1.

### 3.3. Inter-Observer Agreement

**Figure 4:**
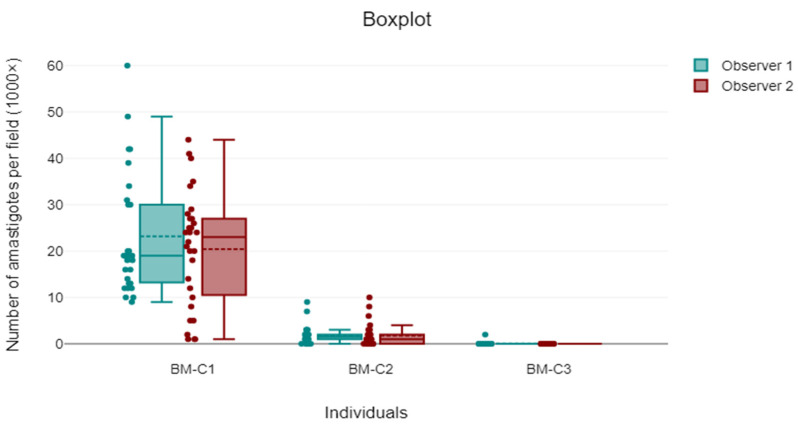
Comparative boxplot showing the distribution of *Leishmania* amastigote counts per 1000× field by Observer 1 (turquoise) and Observer 2 (red) across three individuals (BM-C1, BM-C2, and BM-C3). Each point represents one of the 60 fields analysed per observer and individual. The figure highlights inter-observer variability, particularly in samples with higher parasite burden.

Cohen’s kappa coefficient was calculated to assess the categorical agreement between the two observers in classifying parasite burden. The kappa value was 0.47 (SE = ±0.09; 95% CI: 0.31–0.64; *p* < 0.001), indicating moderate agreement in categorical amastigote classification. The intraclass correlation coefficient (ICC) for continuous counts was 0.63 (95% CI: 0.49–0.74; *p* < 0.001), reflecting substantial agreement. Figure 4 illustrates the agreement between observers, showing the distribution of amastigote counts for each individual.

**Figure 5:**
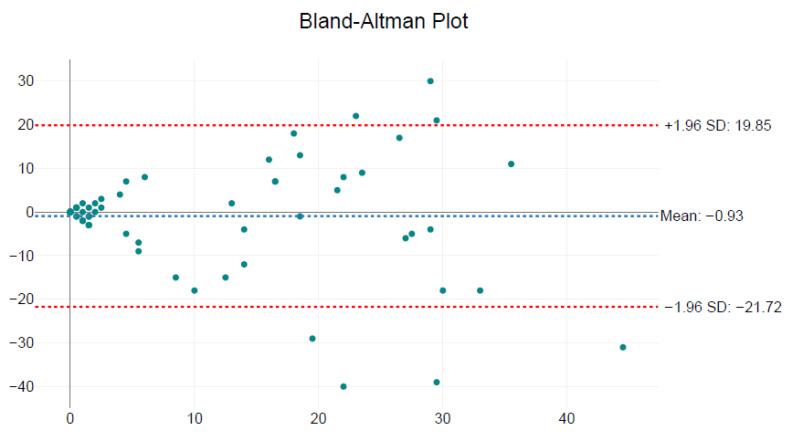
Bland–Altman plot comparing *Leishmania* amastigote counts between Observer 1 and Observer 2 across 60 microscopic fields. The mean difference between observers was −0.93 amastigotes/field (blue dashed line), with 95% limits of agreement ranging from −21.72 to +19.85 (red dashed lines). The data show no systematic bias, supporting acceptable consistency between observers.

Pearson’s correlation confirmed a moderate linear association (r = 0.63; *p* < 0.001). Bland–Altman analysis revealed a mean difference of −0.93 amastigotes/field (limits of agreement: −21.72 to +19.85), indicating the absence of systematic bias between observers (Figure 5).

### 3.4. Correlation Between Parasite Load and Ct Values

A robust inverse correlation was observed between estimated parasite burden and qPCR Ct values (Spearman’s ρ = −0.89; *p* < 0.001). A logarithmic regression model was developed to estimate parasite concentration (amastigotes/μL) from Ct values, according to the following Equations (4) and (5):

(4) $$\log_{10}(Amastigotes/µL)=-0.158\times Ct+7.61,$$

(5) $$Amastigotes/µL=10^{\left(-0.158\times Ct+7.61\right)},$$

**Figure 6:**
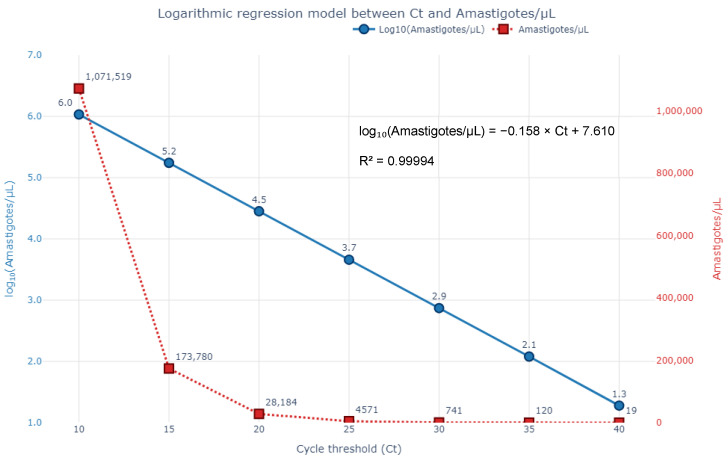
Logarithmic regression model illustrating the inverse relationship between qPCR cycle threshold (Ct) values and *Leishmania* spp. parasite burden in the three bone marrow samples. The blue line (left axis) represents log_10_-transformed amastigote counts per µL, while the red dashed line (right axis) displays the corresponding absolute parasite concentrations. The fitted equation shows excellent correlation: log_10_(Amastigotes/µL) = −0.158 × Ct + 7.610 (R^2^ = 0.99994), supporting the potential use of qPCR for semi-quantitative estimation of parasite burden.

This model demonstrated excellent fit (R^2^ = 0.99994), in this small dataset allowing semi-quantitative estimation of *Leishmania* spp. load from qPCR data. Lower Ct values corresponded to exponential increases in parasite burden, suggesting that qPCR could potentially be used not only for detection but also to inform clinical management based on parasite burden estimates, pending further validation. The fitted curve and corresponding data points are shown in Figure 6. Because parasite counts are markedly skewed and extend across several orders of magnitude, a base-10 logarithmic transformation was applied. This transformation linearises the expected inverse relationship between Ct values and target quantity in qPCR, while also stabilising variance, thereby ensuring that the log-linear model (Equations (4) and (5)) is both biologically meaningful and statistically robust. The three paired observations underpinning Equations (4) and (5) are presented in Supplementary Figure S1.

Based on the logarithmic regression model derived from the relationship between qPCR Ct values and parasite burden determined by direct microscopic quantification in bone marrow smears, a strong inverse association was identified. Specifically, increasing Ct values corresponded to an exponential decrease in the number of *Leishmania* amastigotes per microlitre (µL) of bone marrow. The estimated values, calculated from the regression equation below, are summarised in Table 4.

## 4. Discussion

This pilot study identified an inverse correlation between parasite burden and qPCR Ct values. However, given the small sample size, this finding remains preliminary and requires further validation. The main contribution of this work is the derivation and full reporting of a transparent Ct-to-parasite-burden conversion equation from paired bone marrow cytology and qPCR, including the volumetric assumptions used to transform microscopic field counts into amastigotes/µL. This quantitative bridge between cytology and molecular readouts, supported by an explicit workflow and inter-observer assessment, constitutes the differential of the present study and provides a reproducible starting point for multi-centre validation.

Compared with calibrations based on promastigote or axenic amastigote cultures, or plasmid DNA copy standards, our bone marrow-anchored approach offers greater biological interpretability and matrix relevance. By linking Ct to the correct life stage in the clinical matrix (intracellular amastigotes within canine bone marrow), it inherently incorporates extraction and inhibition effects present in the tissue. Estimates are reported directly as amastigotes per microlitre rather than DNA copies. In contrast, culture- or plasmid-based curves, although convenient and scalable, quantify targets in idealised buffers and require additional assumptions (e.g., copy number per parasite, extraction yield, and matrix corrections), which introduce potential errors. Previous studies have shown that bone marrow (and lymph node) generally yields higher detection rates than peripheral blood and that molecular burden tracks clinical severity in CanL, reinforcing the clinical utility of tissue-anchored calibrations [27,28,29].

Parasite burden was modelled as the mean per HPF rather than as whole-slide totals. This choice improves reproducibility and accessibility, as it can be implemented with standard light microscopy without requiring whole-slide digitisation. It also provides a standardised unit (amastigotes/µL) through a fixed field-to-volume mapping, facilitating comparison across observers and laboratories. Inter-observer analyses confirmed the reliability of HPF-based quantification in this pilot context.

The observed logarithmic regression model yielded an excellent fit across the range of parasite burdens represented in this study, supporting the potential utility of real-time PCR as a semi-quantitative proxy for parasite load in vivo. Lower Ct values, reflecting higher DNA concentrations, were consistently associated with greater microscopic counts of amastigotes. In our dataset, the dog with the highest parasite burden (BM-C1) presented more severe clinical signs, whereas the dog with the lowest burden (BM-C3) was only mildly affected. This pattern aligns with published evidence that higher parasite loads are typically associated with more severe disease [27,28,30,31,32].

The inverse relation between Ct and the target nucleic acid is a foundational principle of quantitative PCR, and not a novel hypothesis. Our exploratory aim was to examine whether qPCR-detected *Leishmania* kinetoplast DNA in bone marrow reflects the cytologically observed amastigote burden. This correspondence is not guaranteed a priori: qPCR detects DNA irrespective of organism viability or cellular localization. Residual kinetoplast DNA can persist transiently after parasite death or treatment. Parasites may be inconsistently distributed within tissues, so that cytology and qPCR aliquots sample different local densities, and extraction/fragmentation differences can alter the amount of amplifiable template [9,11,15,16,17,19,21,23,24,27,28,30].

The high analytical sensitivity of qPCR proved particularly valuable in subclinical or less severe presentations, where parasite burden may be low or focally distributed. In this study, molecular detection identified parasite DNA in cytologically negative bone marrow samples. These findings are consistent with previous reports [28,30], indicating that subclinically infected dogs may still carry detectable levels of parasitic DNA, which frequently escape cytological and serological detection. Furthermore, evidence from comparative studies [33] has established that qPCR outperforms both cytology and culture in sensitivity, especially in tissues characterised by focal or heterogeneous parasite distribution, such as lymph nodes, spleen, and skin. Similar findings [34] were reported using spleen samples from infected dogs, where qPCR demonstrated complete concordance with droplet digital PCR (ddPCR), thereby reinforcing its reliability as a quantitative tool in veterinary diagnostics. In this context, the integration of technological tools such as digital cytology platforms, artificial intelligence-based image analysis, and automated parasite quantification systems may significantly improve diagnostic objectivity and reproducibility [31].

Cytology remains a rapid and cost-effective diagnostic method, but its sensitivity is limited by sampling variability, uneven parasite distribution, and observer-dependent interpretation. In this study, inter-observer agreement was moderate, consistent with previous investigations [29,33].

A key contribution of this work is the derivation of a predictive logarithmic equation allowing estimation of amastigote density per microlitre of bone marrow from Ct values. Although the cytology–qPCR correlation was strong in bone marrow, future studies should evaluate whether similar relationships exist in less invasive matrices such as blood, conjunctival swabs, or skin aspirates [31,34]. While this mathematical model provides a practical framework for translating molecular outputs into biologically and clinically meaningful estimates of parasite burden, its evidentiary basis remains limited. Despite the excellent fit, it should be interpreted cautiously and considered hypothesis-generating and proof-of-concept rather than clinically validated. Comparable approaches have been successfully applied in Chagas disease [35] and CanL [28], and our findings corroborate their relevance. Higher parasite loads were also associated with more severe clinical presentations, in line with established evidence linking parasite density with immunopathology and systemic progression [31].

Nonetheless, some limitations of the present study must be acknowledged. The small sample size reflects the ethical and logistical difficulties of obtaining bone marrow aspirations from client-owned animals, particularly those in early clinical stages. Nevertheless, the inclusion of dogs across a broad parasitic load spectrum enabled meaningful statistical analysis and mathematical model derivation. Previous findings [28] have shown no statistically significant differences in parasite burden among bone marrow, lymph nodes, and spleen (*p* = 0.518), indicating that less invasive sampling sites, such as lymph node aspirates or peripheral blood, may represent viable alternatives for both detection and parasite burden quantification. Future studies should evaluate the reproducibility of our predictive model in these matrices and across diverse epidemiological settings.

From a One Health perspective, early detection and accurate quantification of *L. infantum* in dogs, the primary domestic reservoir, is essential for the control of zoonotic transmission. The integration of qPCR into diagnostic algorithms may enhance the early identification of infected animals, facilitate prognostic stratification based on molecular burden, and enable longitudinal monitoring of treatment response. As demonstrated by Quaresma et al. [29], animals with higher tissue parasite loads often display histological features associated with immune dysfunction, suggesting a possible role for qPCR-derived burden estimates in tailoring individualised therapeutic approaches, strongly endorsed by the LeishVet guidelines [33,36,37]. As highlighted in previous studies [38], persistently high parasite loads at days 28 and 56 post-treatment initiation may serve as early indicators of therapeutic failure or continued transmission risk, underscoring their potential utility as biomarkers for monitoring treatment efficacy in clinical trial settings.

These findings support integrated molecular diagnostics and call for multicentre validation of this model across tissue types, clinical stages, and endemic settings. Future studies should include dedicated validation steps, using serial dilutions of parasite DNA or samples with known parasite counts, to confirm that the observed Ct-amastigote relationship holds true quantitatively. Such integration is consistent with the principles of precision medicine, offering a more refined approach to clinical management and enabling tailored therapeutic interventions based on a comprehensive assessment of infection status. In practical terms, an optimised diagnostic pathway could begin with cytological analysis of readily accessible samples, such as lymph node aspirates, for rapid preliminary evaluation, followed by qPCR confirmation and parasite burden quantification using the same or a complementary sample.

Ultimately, improved diagnostic accuracy benefits affected dogs and supports public health measures. As a preliminary investigation led by a multidisciplinary team spanning veterinary and human medicine, and bioengineering, this study provides foundational data for future validation studies, reinforcing the importance of standardised, evidence-based protocols for diagnosis, monitoring, and control of canine leishmaniosis within a One Health perspective that recognises the interdependence of animal, human, and environmental health.

### Limitations

The pilot nature limits statistical precision and precludes formal validation. The conversion relies on the following: (i) uniform smear distribution and volumetric assumptions; (ii) genus-level target detection with plasmid standards rather than cultured parasites; (iii) potential tissue heterogeneity in parasite distribution. As such, the equation should be regarded as a proof-of-concept and used only for hypothesis generation until validated in larger, representative samples and, ideally, across multiple matrices and laboratories.

Additionally, the extracted DNA was not quantified, and no normalisation to total DNA mass or a canine host single-copy gene was undertaken prior to qPCR. Future validation should incorporate DNA quantification and host–gene normalisation to reduce matrix-related variability and to improve inter-sample comparability.

## 5. Conclusions

Although this was a small-scale pilot, our findings demonstrate a strong inverse correlation between qPCR Ct values and parasite burden in canine bone marrow, consistent with the hypothesis that lower Ct values, even when calibrated using plasmid-based standards, accurately reflect higher in vivo concentrations of *Leishmania* spp. The derived conversion formula provides a preliminary means of estimating parasite burden from molecular data, with potential translational value for clinical decision-making. Given the pilot nature of this study, the findings lay the groundwork for larger investigations to validate the Ct-value conversion model and assess its applicability to early diagnosis, therapeutic monitoring, and integration with automated cytological analysis to minimise observer bias, prior to proposing any modifications to diagnostic or therapeutic protocols. By integrating cytological and molecular approaches, this strategy enhances diagnostic precision, aligns with the principles of precision veterinary medicine, and supports the One Health framework by potentially improving canine disease control and, by extension, protecting human health in endemic regions.

## Figures and Tables

**Table 1:** Thermal cycling conditions used for the detection of *Leishmania* spp. in bone marrow samples.

Cycles	Temperature	Time	Notes
1	95 °C	2 min	Polymerase activation
40	95 °C	5 s	Denaturation
	60 °C	30 s	Annealing/Extension

**Table 2:** Quantification of *Leishmania* amastigote counts per high-power field (HPF; 1000×) by observer and case. For each dog, three smears from the same bone marrow sample were analysed. On each smear, 20 non-overlapping HPFs were counted per observer (N = 60 HPFs per case per observer). Values shown are pooled descriptive statistics across the 60 HPFs per case and observer.

		*Leishmania* Amastigotes per High-Power Field (HPF; 1000×)				
Observers	Samples	Mean	Std. Deviation	Minimum	Maximum	95% CI
Observer 1	BM-C1	23.17	12.84	9	60	18.37–27.96
	BM-C2	1.77	1.96	0	9	1.04–2.50
	BM-C3	0.07	0.37	0	2	0.00–0.20
Observer 2	BM-C1	20.43	12.34	1	44	15.82–25.04
	BM-C2	1.77	2.42	0	10	0.86–2.67
	BM-C3	0.00	0.00	0	0	0.00–0.00

CI, confidence interval; HPF, high-power field (1000×).

**Table 3:** Estimated parasite burden (amastigotes/µL) derived from pooled cytological counts (N = 60 HPFs per case per observer) and corresponding qPCR Ct values for each case.

Samples	Mean Amastigotes ± SD	Ct (qPCR)	Volume per Field (µL)	Estimated Amastigotes/µL
**BM-C1**	21.78 ± 12.56	19.54	0.000607	35,877
**BM-C2**	1.77 ± 2.18	26.00	0.000607	2817
**BM-C3**	0.03 ± 0.26	36.59	0.000607	49.4

**Table 4:** Estimated *Leishmania* spp. burden as a function of qPCR Ct based on the logarithmic conversion model derived from paired cytology. Values are expressed both as base-10 logarithms and as approximate absolute concentrations (amastigotes/µL). Corresponding plasmid copy numbers used as molecular quantification standards are included for reference.

Ct	log_10_(Load)	Estimated Amastigotes/µL	Estimated Plasmid Copies/µL
**10**	6.03	~1.07 × 10^6^	10^7^
**15**	5.24	~1.74 × 10^5^	10^6^
**20**	4.45	~2.82 × 10^4^	10^5^
**25**	3.66	~4.57 × 10^3^	10^4^
**30**	2.87	~741	10^3^
**35**	2.08	~120	10^2^
**40**	1.29	~19	10^1^

## Data Availability

The original contributions presented in this study are included in the article/Supplementary Material. Further inquiries can be directed to the corresponding authors.

## Supplementary Materials

The following supporting information can be downloaded at https://www.mdpi.com/article/10.3390/microorganisms13092211/s1, Table S1: Demographic, clinical, cytological, and qPCR data from canine bone marrow samples. Figure S1: Estimated parasite burden (amastigotes/µL) as a function of qPCR cycle threshold (Ct) in canine bone marrow.

## Abbreviations

The following abbreviations are used in this manuscript:

Ac	area of one microscopic field at 1000× magnification (µm^2^)
A	total estimated slide area (cm^2^)
CanL	canine leishmaniosis
Ct	cycle threshold
C	concentration of amastigotes per microlitre (amastigotes/µL)
EDTA	ethylenediaminetetraacetic acid
FN	field number (ocular lens specification)
HPF	high-power field
ICC	intraclass Correlation Coefficient
IEC	internal Extraction Control
kDNA	kinetoplast DNA
MGG	May-Grünwald Giemsa (stain)
Nc	number of theoretical microscopic fields
NTC	nuclease-free water
PCR	polymerase chain reaction
qPCR	quantitative real-time polymerase chain reaction
R^2^	coefficient of determination
Vc	volume per microscopic field (µL/field)
µL	microlitre
µm^2^	square micrometre
