# Supplementary material for: When Precision Matters: Bone Marrow Cytology Meets qPCR in a Pilot Study Quantifying Leishmania infantum Load in Dogs
Source: Microorganisms. 2025 Sep 21;13(9):2211. doi: 10.3390/microorganisms13092211 (PMC12472918; doi:10.3390/microorganisms13092211)
Supplement: Supplementary file 1 [file microorganisms-13-02211-s001.zip › microorganisms-3758072-supplementary.pdf]

Table S1. Demographic, clinical, cytological, and qPCR data from canine bone marrow samples.

Case ID	Breed	Age (years)	Sex	qPCR Ct (*Leishmania* spp.)	IEC Cq	Cytology (mean amastigotes/HPF ± SD)	Estimated Amastigotes/μL	Clinical notes
**BM-C1**	Dobermann	4.5	Male	19.54	31.821	21.78 ± 12.56	35,877	Vaccinated; dewormed; no concomitant disease
**BM-C2**	Samoyed	3.0	Male	26.00	32.878	1.77 ± 2.18	2,817	Vaccinated; dewormed; no concomitant disease
**BM-C3**	Weimaraner	6.0	Male	36.59	27.334	0.03 ± 0.26	49.4	Vaccinated; dewormed; no concomitant disease

Ct, cycle threshold; IEC, internal extraction control; Cq, quantification cycle; HPF, high-power field (1000×); SD, standard deviation; μL, microlitre

Figure S1. Estimated parasite burden (amastigotes/μL) as a function of qPCR cycle threshold (Ct) in canine bone marrow.

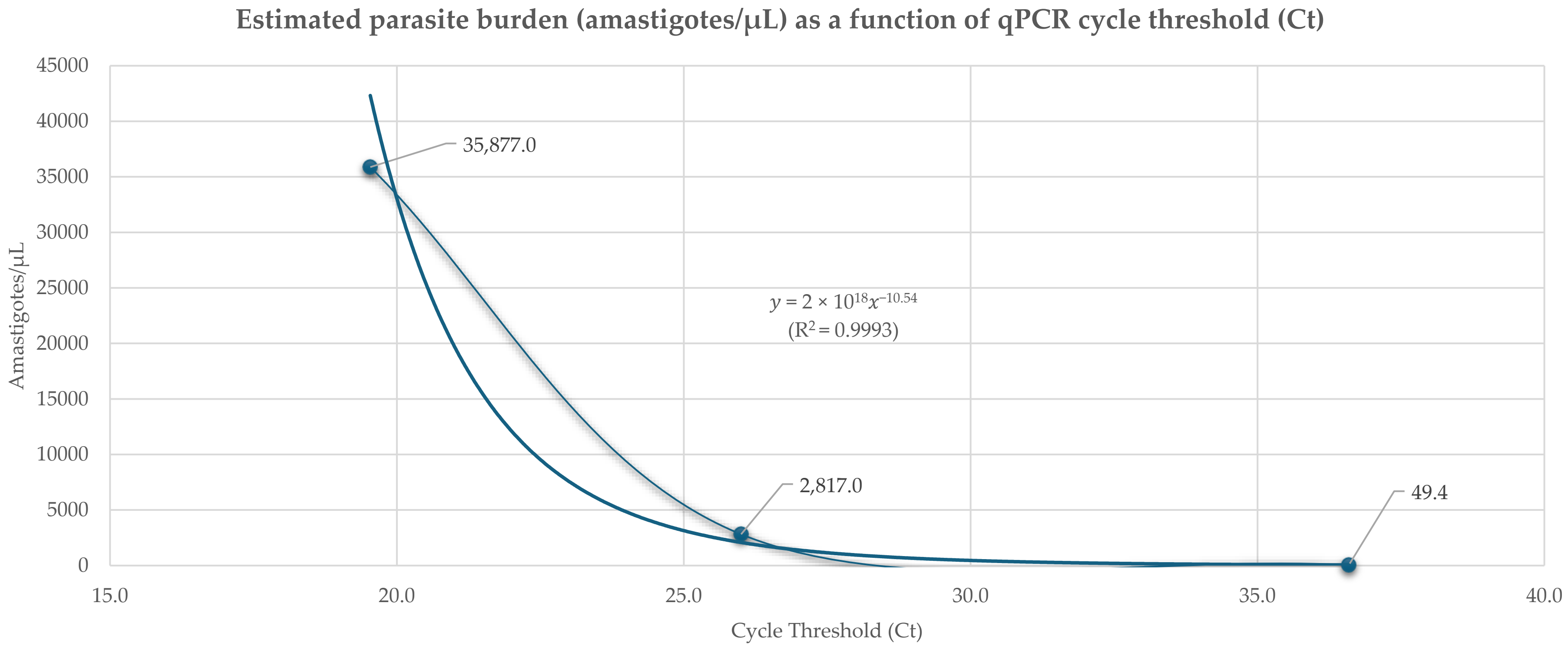

y, parasite burden (amastigotes/μL); *x*, qPCR cycle threshold (Ct).
